# The Impact of Introducing Midwives and also Mentoring on the Quality of Sexual, Reproductive, Maternal, Newborn, and Adolescent Health Services in Low- and Middle-Income Countries: An Integrative Review Protocol

**DOI:** 10.3390/mps6030048

**Published:** 2023-05-05

**Authors:** Rondi Anderson, Sojib Bin Zaman, Mark Limmer

**Affiliations:** 1 The Faculty of Health and Medicine, Lancaster University, Lancaster LA1 4YW, UK; 2 Department of Medicine, School of Clinical Sciences at Monash Health, Monash University, Melbourne 3800, Australia; 3 Centre for Health Inequalities Research, Division of Health Research, Lancaster University, Lancaster LA1 4YW, UK

**Keywords:** midwives, mentoring, maternity care, quality, health systems strengthening

## Abstract

Introduction: Midwives have the potential to significantly contribute to health-delivery systems by providing sexual, reproductive, maternal, newborn, and adolescent health (SRMNAH) care. However, scant research finds barriers to understanding what midwives need to realize their full potential. There are gaps in the definition of a midwife and an understanding of effective means to support the implementation of midwifery care. Mentorship has been found to support systems and healthcare providers to improve care availability and quality. Objectives: We describe the methodology of an integrative review that aims to generate evidence of the impact of introducing midwives and also on-site facility mentoring to better understand facilitators and barriers to implementation of the quality and availability of SRMNAH services in low- and middle-income countries (LMICs). Methods: The Preferred Reporting Items for Systematic Reviews and Meta-Analyses (PRISMA) guidelines will be used to carry out the integrative review. Four electronic bibliographic databases, PubMed MEDLINE, EMBASE, Scopus, and CINAHL, will be used to identify eligible studies. All types of qualitative or quantitative studies will be considered. Eligible studies will be screened according to Population, Intervention, Comparison, and Outcome (PICO) inclusion criteria, and data will be extracted against a predetermined format. The aspects of health system strengthening in providing improved SRMNCH care will be examined in this review to generate evidence on how midwives and mentorship can improve routine care and health outcomes using the World Health Organization’s Six Building Blocks approach. The quality of the articles will be thematically analyzed in four areas: coherence and integrity, appropriateness for answering the question, relevance and focus, and overall assessment using the Gough weight-of-evidence framework. Expected results: The literature review will consider assessing both upstream health systems regulators and downstream effectors for implementing midwifery interventions. Within this building block framework, this research will report on the outcomes and experiences of introducing midwives and the effectiveness of mentoring midwives and other staff in midwives’ roles in improving care quality and health outcomes.

## 1. Introduction

### 1.1. Background

The adoption of evidence-based interventions to improve sexual, reproductive, maternal, newborn, and adolescent health (SRMNAH) remains an international priority under the 2030 agenda for sustainable development [1]. There is also a promise to adopt and scale up priority interventions for increasing access to SRMNAH and protecting human rights, especially for women and adolescents, among the country leaders to attain the 1994 International Conference on Population and Development (ICPD) Programme of Action [2]. Globally, there is a consensus that investing in midwives can be a sustainable, economical way to improve SRMNAH in low- and middle-income countries (LMICs) [3,4]. However, the available evidence sheds limited light on the effective integration of midwifery into LMIC health systems [5]. This is especially true regarding the lack of elements required to enable an environment that defines the position and support of midwives within health systems [6]. It is widely assumed that pre-service education and supportive regulation are sufficient on their own to enable midwives to practice to their full competencies.

A significant challenge, however, in LMIC is transforming the weak SRMNAH service delivery and ensuring its quality [7]. However, the lessons learned from previous attempts have proven that even though globally standard midwives increase quality, integrating full-scope midwifery demands a shift in the system [8]. In order for midwives to achieve their full potential, they may need deliberate support in strengthening their enabling environments. Much is known about the reluctance of existing health systems to change [9]. There has been evidence that low quality, and at times harmful, health care is perpetuated in high-income countries as well as LMICs despite clear guidelines from the World Health Organization (WHO) [10]. Furthermore, studies reveal that midwives have low status within social hierarchies due in part to unequal gender norms. This is evidenced by unsupportive behaviour by facility managers and other maternity staff, poor compensation and security, and demands to comply with medical practices that are not supported by evidence [11]. Based on these findings, inputs are needed to ensure supportive working conditions for newly introduced midwives in order for them to fulfill their potential.

As LMICs strive to ensure quality sexual and reproductive health care, mentorship is increasingly being promoted. Previous research indicates that facility mentorship provides a wide range of positive outcomes (e.g., increased confidence, commitment, and motivation) [12,13]. In addition, mentees reported gaining knowledge and professional competencies [14]. It is found to be more effective than training alone, but it is applied less frequently [15]. Mentorship includes both clinical and facility-wide activities designed to create enabling environments and enhance the capacity of both management and clinicians [16]. Through observation and constructive feedback, mentors help to strengthen existing systems for midwives [17]. However, facility mentoring has constraints, including a shortage of skilled human resources, limited availability of experienced local mentors, and the need to provide ongoing mentoring support as opposed to one-time training [18,19,20]. It is essential for facility-wide activities to incorporate education and buy-in from facility managers and other healthcare service providers (e.g., doctors and nurses) regarding the roles of midwives and evidence-based SRMNAH practices [8,21]. 

### 1.2. Objectives

The purpose of this literature review is to examine programs that have introduced midwives and also mentoring in LMICs to better understand facilitators and barriers to the provision of quality SRMNAH services. We hypothesize that introducing midwives to enabling environments improves the quality of SRMNAH care in LMICs. Our secondary hypothesise is that introducing midwives with mentoring can positively impact the building blocks of health systems related to the improvement of SRMNAH care. In fact, strengthening all the building blocks is essential for successful sustainable implementation. This study seeks to provide insight into various effective methods for improving health systems in LMICs. In addition to providing insight into how midwives with enabling environments can facilitate the implementation of evidence-based care and improve health outcomes, this review article includes articles on the experiences of facility managers and healthcare providers on facility mentoring. The facility mentoring in this review aligns with the WHO quality maternal health network guidelines, which delineate on-site support, learning and sharing, measurement, and community and stakeholder engagement. Governments and development partners will be supported to design more effective programs for improving the availability and quality of SRMNAH in low-resource settings if our knowledge regarding this topic is refined.

### 1.3. Rationale

There is a global movement to increase the number of midwives in LMICs. In spite of this, there are still gaps in the understanding of how to enable midwives to their full potential [22,23,24]. In addition, midwives face barriers, including gender and power dynamics within health systems [11]. Because of these barriers, the impact of professional midwives in providing SRMNAH services is not well documented in LMICs. As outlined above, health facility mentoring can be an effective intervention to guide managers, providers, and facility systems to adhere to standard procedures to maintain high-quality services [25,26,27]. Although the WHO endorses facility mentoring, there is a lack of research to evaluate the process and effectiveness of mentorship on midwives to provide SRMNAH services in LMICs.

Until now, no systematic or integrative review has been conducted to summarize findings regarding the impact of midwives and mentoring on SRMNAH services. In order to answer the review question, it would be appropriate to conduct an integrative review approach, in which the authors would be able to combine the analysis of both qualitative and quantitative studies [28]. The inclusion of qualitative studies for conducting an integrative review would be more impactful than a systematic review for this type of review [29]. This review aims to generate evidence regarding the impact and experience of deploying midwives and mentorship programs in SRMNAH in LMICs on all aspects of administering and providing midwifery care.

## 2. Methods

The protocol of this systematic review is registered in PROSPERO (CRD42022367657).

### 2.1. Study Design

This integrative review will be conducted using the Preferred Reporting Items for Systematic Reviews and Meta-Analysis (PRISMA) guideline [30] and adopting Arksey and O’Malley’s framework [31]. This framework provides five stages to complete this review: (i) identifying the research question; (ii) identifying relevant studies; (iii) study selection; (iv) charting the data; and (v) collating, summarizing, and reporting the results [31], which we consider to present our findings. Since the data synthesis will be based on publicly available data (published articles), we do not intend to seek ethical approval from an institutional review board.

We found some useful frameworks to analyze our findings, such as the WHO Health Systems Building Block Framework, the WHO health labor market framework, a quality-of-care framework, and the Renfrew et al. framework from the Lancet series on midwifery. We will use the ‘Health Systems Building Block Framework’ for this review to compare and contrast studies with or without mentoring support for midwives.

All of these frameworks are potentially helpful and relevant. However, we found ‘Health Systems Building Block Framework’ are more helpful in assessing the process of strengthening health systems by introducing midwives with or without mentoring support. In order to better understand how interventions can work in complex, real-life settings, the WHO has recommended the use of a health systems ‘building block’ framework, such as (i) service delivery; (ii) health workforce; (iii) health information systems; (iv) access to essential medicines; (v) financing; and (vi) leadership/governance. As part of this process, we will examine the six WHO building blocks that define the essential components of health systems.

### 2.2. Eligibility Criteria

The review will be guided by the Population, Intervention, Comparison, and Outcome (PICO) framework [32,33]. We will use this instrument for the selection and screening of articles to be included in the systematic review. Table 1 provides the key concepts and search terms that will be used for the screening of articles. The below inclusion criteria will be applied following the PICO framework:

- Population: Midwives, maternity staff (nurses, paramedics), health managers, patients receiving care
- Intervention: Midwifery-led interventions with mentorship on the quality of maternal and newborn healthcare
- Comparison: Effective vs. ineffective for health system strengthening and quality and availability of services
- Outcome: Operationalisation of evidence-based maternal health care within health systems, improved health outcomes including health and well-being of women and newborns, experiences of midwives, facility staff, and managers regarding deploying midwives, and improvements in care quality.

**Table 1:** Search terms used for the literature.

Key Concepts	Search Terms
Midwives/midwifery	Delivery, Obstetric; maternal health services; midwife * or midwiv *; maternal; skilled birth attendan *
Enabling environment	Enabling environment; supportive environment; mentoring; mentor; supervis *
Care quality/care improvement	Quality of health care; quality improvement; care quality; outcome; quality improvement; healthcare
Low- and middle-income countries (LMICs)	All countries will be included as per World Bank’s definition

The asterisk (*) has been used as a wildcard character to represent one or more unknown characters in a search query.

### 2.3. Exclusion Criteria

This review will exclude case reports, ideas, editorials, meta-analyses, protocol papers, review articles, and opinions. The following criteria will be followed to exclude:

(i) articles or reports published before 2010;
(ii) national surveys published between 2010 and March 2023;
(iii) studies that do not provide any impact or outcome;
(iv) articles that are not available in full text; or
(v) articles that are not accessible after contacting the corresponding author.

### 2.4. Study Selection/Type of Study to Be Included

All types of qualitative or quantitative studies will be considered. In addition, we will include randomised trials to assess the beneficial effects of midwifery-led care and supplement these with cross-sectional and case-control studies to determine the impact. We will include the eligible articles between 2010 and March 2023. While conducting a literature search from their inception till to date will be the ideal timeline; however, we intend to include only the published articles over the last decade to gather current practices and evidence related to midwifery care. The country where the study was conducted will not influence the selection of articles.

### 2.5. Search Strategy

Predetermined major concepts (i.e., midwives/midwifery, supervision/mentoring, and care quality/care improvements) will be searched with specific subject headings and the related Medical Subject Headings (MeSH) or thesaurus terms (Table 1). For example, we will use search terms of ‘adolescent’ or ‘sexual’ or ‘reproductive health’ or ‘abortion’ or ‘pre-martial sexual behavior’ or ‘child marriage’ or ‘childbearing’ or ‘maternal health’ or ‘family planning’ or ‘sexually transmitted infections’ or ‘cervical cancer’ or ‘menstrual regulation’ or ‘gender-based violence’ or ‘bodily autonomy’ or ‘harassment’ and ‘delivery’ or ‘obstetric’ AND ‘midwife*’ or ‘midwiv*’ or ‘maternal’ or ‘nurse’ or ‘skilled birth attendan*’ AND ‘enabling environement’, ‘mentoring’ or ‘mentors’ or ‘mentorship’ or ‘supervis*’ AND ‘quality of health care’ or ‘quality improvement’ or ‘care quality’ or ‘outcome’ or ‘quality improvement’ or ‘healthcare’ AND ‘LMICs’ or ‘low-middle-income countries’ or any country as per World Bank definition under the LMIC category. Expert librarians from Lancaster University will be consulted to refine and finalize the search strategy.

Reference lists from important articles will be scanned to identify other relevant articles not found in the initial search. Articles identified in a manual search of relevant journals and references related to midwifery and SRMNAH in LMICs will be reviewed from the retrieved papers. In case of unavailability of this article, the online study team will be contacted via email, and discussions will be held by phone or in-person to ask about any recent relevant publications.

### 2.6. Information Sources

Articles published in English from 2010 to July 2022 for this review will be considered. Since the concept of professional midwives has gained popularity in recent years, we are not considering the literature search before 2010. It is well established that reviews from the past ten years are thought to capture current contexts and issues [34]. We will use PubMed MEDLINE, EMBASE, Scopus, and CINAHL electronic bibliographic databases for searching relevant articles. In addition to the database search, internet searches of published reports and grey literature will be conducted. Hand-searching pertinent reference lists will be considered using a snowball approach if required. 

### 2.7. Data Extraction (Study Selection and Coding)

All articles obtained from the selected databases will be exported to EndNote (version X8.2, Clarivate Analytics), and exact duplicates will be removed. Two reviewers will screen the title and abstract independently for eligibility using the predefined inclusion and exclusion criteria. Conflicts will be resolved by discussion with a third reviewer. Reasons for exclusions will be recorded. Study investigators will be contacted for unreported data or additional details. Data will be recorded in an Excel spreadsheet. Table 2 summarises the information that we aim to collect.

**Figure 1:**
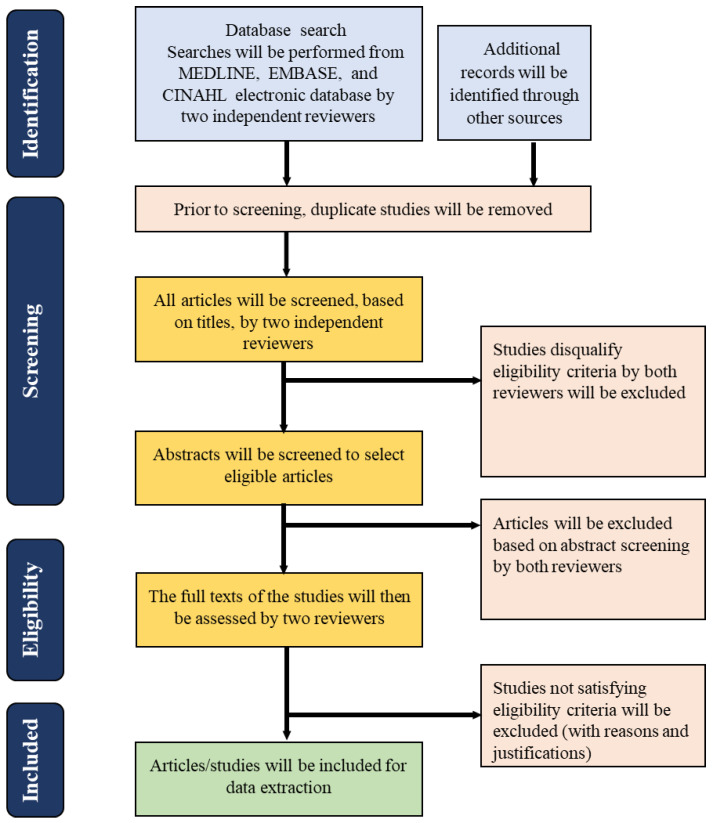
PRISMA flowchart for the screening and selection of studies.

All articles meeting the inclusion criteria will be exported to a Microsoft Excel spreadsheet, and the two reviewers will independently extract the data. Using a predefined and pretested data extraction Excel spreadsheet, the following information from each relevant article will be extracted: first author, country, year, title, study aim, study methods, participants, themes, use of professional midwives, mentoring, and critical findings. Figure 1 shows the process of screening and reviewing abstracts and full-text articles to be followed based on the eligibility criteria.

#### Operational Definition

**Midwife:** A midwife is a health care professional who has completed a midwifery education program based on the ICM Global Standards for Midwifery Education and is recognized by the country where it is conducted.

**Midwifery-led intervention:** A midwife-led model of care entails the midwife being the primary healthcare practitioner responsible for the planning, organization, and delivery of care given to a woman from the initial scheduling of antenatal appointments through postnatal care.

**Effective intervention:** An intervention is effective if it successfully produces the desired or intended outcome.

**Outcome and impact:** While outcome refers to particular and measurable short-term consequences of an intervention, the impact can adopt a broader perspective, focusing on larger and longer-term implications.

### 2.8. Data Analysis and Presentation

We will use WHO-derived Health Systems Building Block Framework for extraction, synthesis, and analysis [35]. Data from the articles will be iteratively compared to identify common sub-themes relevant to the research question. Finally, the sub-themes will be coded and aggregated to determine emerging themes according to the Building Block Framework. This will facilitate the comparison of contrasting information within the identified themes (e.g., service delivery, human resources, medicines and technologies, financing, information, and leadership and governance). In addition, this process will allow for systematic organization, analysis, and reporting. Following the analysis, we will narratively report the findings.

### 2.9. Quality Assessment

To assess quality, a combined, modified mixed-methods synthesis tool developed at Leeds University, together with the Gough (2007) weight-of-evidence framework, will be used [36]. The Gough tool guides quality evaluation using four themes: coherence and integrity, appropriateness for answering the question, relevance and focus, and overall assessment (Table 3). Two reviewers will be involved in the quality assessment. The third reviewer will manage the disagreement if it is required.

## 3. Expected Results

This protocol describes the methodologies to conduct an integrative review of providing improved SRMNAH service to strengthen health system readiness through the introduction of midwives in LMICs. Health system strengthening, as defined by WHO, involves enhancing the functioning of the health system across six component areas or building blocks: service delivery, workforce, information systems, access to essential medicines, financing, and leadership and governance [35]. The aspects of health system strengthening will be examined in this review to generate evidence of how midwives and mentorship can improve routine care and health outcomes using the building blocks approach. The literature review aims to provide a broad understanding of current knowledge on the topic and highlight successes and challenges related to professional midwives and mentoring in LMICs.

Creating enabling environments for midwives can play a crucial role in contributing to health financing and health information system. Midwives can play an essential role in improving the health financing system in several ways, including advocacy (e.g., for the allocation of resources to SRMNAH areas with the greatest need), cost-effective care (e.g., to reduce unnecessary medical interventions and lower the overall cost of care), prevention and early detection (e.g., help to reduce the burden on the health system) and community engagement (e.g., to raise awareness for seeking SRMNAH care). In addition, to ensure enabling environments for midwives, facility mentoring can contribute to improving the health information system by collecting accurate and complete data, analyzing data to inform decision-making, using data to improve the quality of care, leveraging technology to improve data collection and management, and providing training and capacity building to enhance the skills of other health workers and community members.

It has been estimated that more investment in midwife-led interventions to provide SRMNAH services could save 4.3 million lives annually [1]. As frontline health workers, midwives provide extraordinary support in promoting health and well-being on SRMNAH [37]. The addition of mentoring facilities by midwives can support managers and midwives in transitioning into their new roles and introduce improved care quality [38].

This review has some limitations. One of the limitations of this review is restricting the literature search between 2010 and March 2023, which could raise the issue of publication bias. However, midwifery care is a new concept for most of the countries in LMICs, so including recent articles for this search could be justifiable. Another limitation could be to consider only four search databases for selecting eligible articles and studies. Given the robustness of the review, it will be better to focus either on the midwifery introduction or on the effect of mentoring on midwives in this integrative review. However, this review will provide a greater comprehensive understanding of a particular phenomenon related to the quality of midwifery care on SRMNSH with an enabling environment.

The literature review will consider assessing both upstream health systems regulators and downstream effectors for implementing midwifery interventions. Within this framework, this research will report on midwives’ experiences in their roles, quality improvements, and health outcomes. It is expected that by further refining our knowledge on this topic, some recommendations will derive which may help to design a more effective program to improve the quality of SRMNAH care in low-resource settings.

## Figures and Tables

**Table 2:** Information that is planned to be collected from individual studies.

Sl.	First Author, Country, Year	Title	Study Aim	Study Methods & Participants	Themes	ICM-Standard Midwives	Maternity Staff	Mentoring	Key Findings
1									
2									
3									
4									

**Table 3:** Description of Gough tool used for this review.

Dimension	Description of Review
A. Coherence and integrity	A generic non-review-specific judgment of the coherence and relevance on its own terms, using the generally accepted criteria for this type of evidence.
B. Appropriateness for answering the question	A review-specific judgment about the fitness for purpose of the evidence for answering the question.
C. Relevance and focus	A review-specific judgment about the relevance of the focus of the evidence for the question. This could include issues of propriety in how the research was conducted, which could impact its inclusion and interpretation.
D. Overall assessment	The three above judgments are then combined to give an overall assessment.

## Data Availability

Data sharing does not apply to this article.
